# The Prince of Wales's Hospital Fund

**Published:** 1900-11-24

**Authors:** 


### THE PRINCE OF WALES'S HOSPITAL FUND.

### AN APPEAL.

Owing to the claims of sufferers from the war, the directors of the Prince of Wales's Hospital Fund suspended their appeal to public support until the present time. The Hon. Secretaries have issued the following letter:—

Bank of England, London, E.C., November 22nd, 1900.

Sir,—Since the outbreak of hostilities in South Africa the executive committee of the Prince of Wales's Hospital Fund have refrained from making any appeal on behalf of the hospitals because of the exceptional claims that have been made upon the public in respect of the War and Famine Funds, but the falling-off in the financial support of the hospitals of London (which is somewhat natural under the circumstances) must be made good if these institutions are to fully continue their useful work.

The executive committee, therefore, appeal to you to assist his Royal Highness the President and the council to make a distribution this year that will materially help to counteract the falling-off that has taken place.

A short statement of the work of this fund is sent herewith, which the executive committee venture to think shows that any moneys entrusted to them will be expended in a manner best calculated to produce the most satisfactory results.

We beg to enclose a subscription form which we earnestly hope you may be willing to fill up for some contribution. It is desirable that further help up to a *minimum* amount of £25,000 may be given before December 15th next.

Yours faithfully,

Duncannon, J. G. Craggs, } Honorary Secretaries.

The latest report of the Hospitals Distribution Committee shows the good work that is done by the fund:—

"The past year has been one of increased usefulness of the fund.

"The hospitals concerned have been again visited by sub-committees, consisting of medical men of wide experience and laymen who take special interest in hospital management. These visitations, while they have admirably answered their immediate object of supplying reliable information regarding the merits and needs of the various institutions, have proved of very high value in stimulating improvements, both in construction and in all departments of administration. Defects have been pointed out, and their remedy has been powerfully promoted by making the grants from the fund conditional upon the carrying out of the measures that have been recommended.

"The suggestions made by the visitors have been almost invariably received in a most friendly spirit, and new interest has been often aroused in those more immediately concerned in the several hospitals, leading them to increase their own subscriptions, or to promote in other ways by their individual exertions the needed improvements.

"The importance of the services rendered by the fund has been more generally recognised by the public, so that the sum available for distribution to hospitals this year shows a considerable increase upon last year's amount; the figures being £41,000 now, as compared with £31,500 in the previous twelvemonth.

"It is anticipated that the League of Mercy, instituted a year ago by his Royal Highness, will in due time greatly benefit the fund; but the committee can at present only advise the employment of £1,000 from that source, this being the amount which had been actually paid in when this year's distribution was arranged.

"It is recommended that the additional £9,500 now at the disposal of the fund be employed partly in the way of donations and partly as annual grants, which are necessary when money is given for the important purpose of opening closed wards, for which continuous maintenance must be provided.

"It has been arranged that 45 beds now unoccupied will be brought into use next year; these, added to those thrown open during the last two years, give 287 as the number made available to the poor of the metropolis by the agency of the fund."

Included in last year's list of subscribers and donors were the following annual subscriptions:—

The Prince of Wales, £105; the Princess of Wales, £25; Princess Victoria of Wales, £5; the Duke and Duchess of York, £21; Princes Albert and Edward of York, £2 2s.; Princess Victoria of York, £1 1s.; the Duke and Duchess of Fife, £50; the Duke and Duchess of Teck, £10; Captain Prince Louis of Battenberg, R.N., £1 1s.; Princess Louis of Battenberg, £1 1s.

In 1897, £56,826; in 1898, £32,500; in 1899, £42,000. To keep up this excellent record the fund must be able to rely upon much more assistance than it has received this year.

The cost of management and collection is about 3¾ per cent.

